# The psychometric properties of the AUDIT: a survey from a random sample of elderly Swedish adults

**DOI:** 10.1186/1471-2458-14-672

**Published:** 2014-07-01

**Authors:** Håkan Källmén, Peter Wennberg, Mats Ramstedt, Mats Hallgren

**Affiliations:** 1 Stad, Centre for Psychiatry Research, Karolinska Institutet, Norra Stationsgatan 69, Stockholm 113 64, Sweden; 2 Department of Public Health Sciences, Division of Social Medicine, Karolinska Institutet, Stockholm, Sweden; 3 Centre for Social Research on alcohol and drugs, Stockholm University, Stockholm, Sweden; 4 Department of Public Health Sciences, Division of Epidemiology and Public Health Intervention Research (EPHIR), Karolinska Institutet, Stockholm, Sweden

**Keywords:** AUDIT, The elderly, Alcohol consumption, Swedish general population

## Abstract

**Background:**

Increasing alcohol consumption and related harms have been reported among the elderly population of Europe. Consequently, it is important to monitor patterns of alcohol use, and to use a valid and reliable tool when screening for risky consumption in this age group. The aim was to evaluate the internal consistency reliability and construct validity of the Alcohol Use Disorders Identification Test (AUDIT) in elderly Swedish adults, and to compare the results with the general Swedish population. Another aim was to calculate the level of alcohol consumption (AUDIT-C) to be used for comparison in future studies.

**Methods:**

The questionnaire was sent to 1459 Swedish adults aged 79–80 years with a response rate of 73.3%. Internal consistency reliability, were assessed using Cronbach alpha, and confirmatory factor analysis assessed construct validity of the Alcohol Use Disorders Identification Test (AUDIT) in elderly population as compared to a Swedish general population sample.

**Results:**

The results showed that AUDIT was more reliable and valid among the Swedish general population sample than among the elderly and that Item 1 and 4 in AUDIT was less reliable and valid among the elderly.

**Conclusions:**

While the AUDIT showed acceptable psychometric properties in the general population sample, it’s performance was of less quality among the elderly respondents. Further psychometric assessments of the AUDIT in elderly populations are required before it is implemented more widely.

## Background

A recent report indicates there are clear signs that alcohol consumption and alcohol related harm have increased among “adults” (>60 years) in Europe [1]. In Sweden, this increase has been paralleled by a convergence in the amount of consumption. Today, per capita, alcohol consumption among people above 65 years is approaching the adult average.-For instance, in 2012 per capita consumption among youth aged 16–19 years and adults aged 65 to 80 years was the same [2,3]. As the elderly have been somewhat neglected in previous studies on drinking, it is important to assess the consumption habits of this age group and in particular to improve our knowledge about what methods are appropriate to screen for alcohol problems among the elderly.

There are many specific challenges to assess drinking habits among the elderly in addition to those that are well known in survey research in general, e.g. underreporting and biased non-response. For instance, there is a high prevalence of health problems among the elderly and also a large proportion living in institutions [4]. In addition to the general problems with obtaining representative samples, other specific difficulties include hearing problems and cognitive impairments [5]. One implication of these difficulties is that any survey assessing drinking habits among the elderly should be relatively simple and short.

One such assessment tool is the Alcohol Use Disorders Identification Test (AUDIT). The AUDIT is an established instrument and has an English manual [6]. This is a 10 item questionnaire that was developed for the purpose of screening risky alcohol consumption. It is reported to be valid and reliable for use in primary care populations [7]. Acceptable reliability and validity has also been demonstrated on a population basis [8]. AUDIT was used to evaluate changes in alcohol habits in the Swedish population during the years 1997, 2001, 2005, and 2009 [9]. Across all surveys the participants were aged between17 and 71. In these four population studies 1997–2009 we estimated the data quality in terms of reliability (Cronbach alpha) and covariance structure (explorative principal axis factoring).

However, there is no study of the psychometric performance of AUDIT on the ages among adults over 71 year in Sweden. In their 2007 update of the review of studies on the AUDIT, Reinert and Allen [10] concluded that AUDIT is not valid among older age groups due to low sensitivity. They suggested a lower cut-off score indicating hazardous drinking and alcohol problems in higher ages. O’Connell et al., [11] also pointed to the low sensitivity of AUDIT to screen for alcohol problems in elderly people. They suggested the use of AUDIT-5, a brief version of AUDIT consisting of items 2, 4, 5, 9 and 10. By using a sub-group of the Finnish population aged 65–74, Aalto et al., [12] suggested a cut-off score of five instead of eight. This result is not necessarily valid for people aged above 74 year but their main conclusion was that AUDIT is still valid for higher ages but at lower cut-offs.

In the present study we investigate the co-variation between items and the factor structure of AUDIT among elderly persons aged 79–80 years and compare the results to the general Swedish population aged 17–71 year. In earlier studies of the factor structure of AUDIT it has been shown that one single factor is valid among heavy drinkers [13] and two factors, consumption (item 1–3) and alcohol problems (items 4–10) are valid among the general population [8]. AUDIT was, however, constructed as a three dimensional test, i.e. consumption, dependency (item 4–6) and harms (item 7–10) [7]. The factor structure is important due to the association between the three dimensions. A single factor structure indicates that respondents strongly associate alcohol consumption with alcohol related problems but a two factor structure indicates a lower association between those constructs. A three factor structure indicates that respondents divide alcohol related problems into dependency and harm. The factor structure therefore indicates the alcohol habits in the surveyed sample.

The purpose of this study is to test the reliability and construct validity of AUDIT when applied in a sample of elderly respondents aged 79 or 80 years and to compare the results to subjects in the general Swedish population aged between 17–71. The hypotheses are that no differences exist between the elderly and the general population in reliability (internal consistency) or validity (factor structure). A second aim was to provide normative data for future comparisons for both AUDIT and the shortened version of the test, AUDIT-c (the three first items of the AUDIT), among elderly persons. A third aim of the study is to investigate if late responders score similar to early responders. On the assumption that late responders are more similar to non-responders in terms of alcohol habits, this could be an indication of alcohol habits in the non-responding group. Due to our earlier studies with AUDIT we anticipated that men would score higher than women and there would be no differences between early and late responders.

## Methods

### Participants

In spring 2013 a postal survey containing the AUDIT questionnaire was sent to 1459 subjects aged 79–80 year (50% women). The response rate was 73.3%, 538 men and 528 women. The participants were randomly sampled from a registry of all people living in Sweden (DAFA/SPAR). The mean age for men was 79.5 years (SD = 0.5) and for women 79.9 years (SD = 0.4). Data collected from the participants were compared to data from 663 men and women who responded to the 2009 survey of the Swedish general population (17–71 years), regarding internal consistency and factor structure. Non-drinkers were included in the analyses. Two outliers were identified and were excluded, and a coding error was corrected.

### Design

The data collection procedure was identical for both the general population and for the elderly sample. After the main mail two reminders were sent by post to non-responders. In the group, just over half the sample (52.2%), responded initially and after the first reminder the response rate was 69.4%. The second reminder increased the response rate to 73.3%. In the population sample from 2009 the response rate was 54% (276 men and 344 women). There were 43 responses without indication of gender. Our assumption, that the late-responders were more similar to the non- responders than to the early-responders was also tested.

Since it is known that the average AUDIT score is higher among men than among women, data was stratified by gender to be able to make gender specific means and standard deviations (Table 1). The Regional Ethical Review Board in Stockholm approved the study (Dnr 213/1086-32).

**Table 1:** Mean (M) and standard deviation (SD) of AUDIT and AUDIT-c for men and women among the elderly (N = 1066)

	**AUDIT**		**AUDIT-c**	
	**M**	**SD**	**M**	**SD**
Men elderly	2.36	2.50	1.99	1.76
Women elderly	1.28	1.63	1.20	1.44
Women control group	3.38	2.59	2.43	1.72
Men control group	4.72	4.20	3.16	2.24

### Statistics

All analyses except the confirmatory factor analysis were performed in SPSS 22.0. Descriptive statistics (means, and standard deviations) show the distribution of data. Internal reliability was evaluated by using Cronbach alpha. The 2 (gender) × 3 (postages) ANOVA tested the significance of differences in alcohol habits between men and women and also between responders on the three postages. The exploratory factor analysis showed the prevailing covariance structure of the data. Items loading above 0.4 on a factor were considered to show a high degree of association to that factor. Missing data were excluded listwise and no imputation was made. The confirmatory factor analysis tested the construct validity of the data and was made in STATA 12.0. In this analysis both genders were included. The fit of the models in the confirmatory factor analyses was expressed in Chi-square, Root mean square error of approximation (RMSEA), Comparative Fit Index (CFI) and as Tucker-Lewis index (TLI). The confirmatory analyses were carried out on both the elderly (79–80 years) and on the general population (17–71 years) samples.

## Results

The internal consistency reliability of AUDIT (Cronbach alpha) was 0.55 (95% CI 0.51-0.59) in the sample of 79–80 year olds respondents. It was significantly lower than the 640 respondents aged 17–71 year investigated 2009 (alpha 0.77, 95% CI 0.75-0.80). The lower reliability among the elderly was probably due to the lower item-total correlations for items 4–10.

Confirmatory factor analyses (Table 2) tested the fit of three models (one, two and three factors) to the covariance structure of the data. Results indicated that all three models were significantly different from the structure of data and the fit was moderate but acceptable for 2 and 3 factor solutions in the population sample (between .05 and .08) but was not satisfactory for the elderly [14]. A comparison of the confidence intervals for the elderly and general populations on each factor structure showed a marginal overlap, suggesting that the construct validity is lower for the elderly. In both samples the two-factor model shown to be valid in earlier studies (consumption (Items 1–3) and alcohol problems (Items 4–10) as latent variables), demonstrated a fit nearly as good as the tree factor model (with consumption, dependency (item 4–6), and “alcohol related harms” (items 7–10) as latent variables). Exploratory factor analyses showed that the misfit of the theoretical factor structures of AUDIT among elderly was due to the low reliability and practical validity (factor loadings below 0.4) [15] of item 1 and 4. There were inconsistencies in the response pattern on these items. For example, some elderly respondents who reported never drinking alcohol on item 1 still scored between 1 and 4 total points. Among those who reported never binge drinking some of the elderly respondents scored as high as 14 points.

**Table 2:** Confirmatory factor analyses of one, two and three factor models of AUDIT

	**1 factor elderly**	**2 factor eldery**	**3 factor elderly**	**1 factor population**	**2 factor population**	**3 factor population**
Chi-square	620.864	406.892	402.118	402.604	250.628	234.649
Df	35	34	32	35	34	32
P	<0.01	<0.01	<0.01	<0.01	<0.01	<0.01
RMSEA	0.126	0.102	0.105	0.109	0.085	0.084
90% CI of RMSEA	0.117-0.135	0.093-0.111	0.096-0.114	0.099-0.118	0.075-0.095	0.07-0.10
CFI	0.746	0.838	0.840	0.865	0.921	0.926
TLI	0.674	0.786	0.775	0.827	0.895	0.896

A comparison between elderly (79–80 years) 2013 (N = 1066) and the Swedish general population (17–71 years) 2009 (N = 640).

The exploratory principal axis factoring with promax rotation based on data from the elderly sample showed that three factors with eigenvalues above 1.0 explained 57.33% of the co-variance between the items. As shown in Table 3 items 2 and 3 loaded on factor one, items 5, 6, 7 and 8 loaded on factor two and items 9 and 10 loaded on the third factor. Items 1 and 4 did not load above .4 in any factor indicating a low correlation between item 1 (How often you drink alcohol) and items 2 and 3 (below 0.3). This also applied to item 4 (unable to stop the drinking after a drinking session). Item 4 showed a low correlation (about 0.2) with items 5–10. Factor 1 correlated 0.31 with factor 3 and 0.57 with factor 2, which in turn correlated 0.51 with factor 3.

**Table 3:** Explorative principal axis factoring loadings of the AUDIT based on the responses from 79–80 years old (N = 1066)

**Item**	**Consumption**	**Dependency**	**Alcohol harms**
1 How often do you have a drink containing alcohol?			
2 How many drinks containing alcohol do you have a typical day when you are drinking?	0.562		
3 How often do you have six or more drinks on one occasion?	0.806		
4 How often during the last year have you found that you were not able to stop drinking once you have started?			
5 How often during the last year have you failed to do what was normally expected of you because of drinking?		0.606	
6 How often during the last year have you needed a first drink in the morning to get yourself going after a heavy drinking session?		0.528	
7 How often during the last year have you had a feeling of guilt or remorse after drinking?		0.655	
8 How often during the last year have you been unable to remember what happened the night before because of your drinking?		0.635	
9 Have you or someone else been injured because of your drinking?			0.659
10 Has a relative, friend, doctor or other health care worker been concerned about your drinking or suggested you to cut down?			0.875

The means and standard deviations of AUDIT and AUDIT-c for both genders are shown in Table 1. Since it is well established that men and women differ in their level of alcohol consumption, data was stratified on gender. An 2 (gender) × 3 (postages) analysis of variance (ANOVA) showed that there was a significant main effect of gender (F(1,1048) = 4.44, p = 0.035), with men scoring higher than women. We found no differences in scores on AUDIT or AUDIT-c between the three mail-outs, indicating that the late responders did not score differently than early responders. This suggests that the non-responders probably did not affect the result.

## Discussion

As earlier research has shown that the elderly are increasing their alcohol consumption and alcohol related harms have also risen it is important to assess alcohol habits in this population and do so with valid methods. The Alcohol Use Disorders Identification Test (AUDIT) was developed to screen alcohol habits in primary care and has been shown to have sufficient reliability and validity among adults aged 17 to 71 years in Sweden [9]. The present study aimed to evaluate the internal consistency reliability and construct validity of the AUDIT-in a Swedish population aged between 79 and 80 years. The results showed that the internal consistency reliability of AUDIT was significantly lower in elderly people aged around 80 and the construct validity, in terms of factor structure, was lower than in the general adult population. Lower reliability was mainly attributable to lower inter-item correlations for the alcohol problems subscale (item 4–10). This might be explained by the fact that in older age people report more varied alcohol consumption due to problems with health and medication. Had we examined the drinkers only, it is possible that a higher degree of reliability would be observed. The low reliability of item 1 and 4 resulted in a lower construct validity and poorer fit to the theoretical models among the elderly compared to the general population. Item 1 concerns how often you drink alcohol. In light of the changing overall drinking habits in Sweden, with alcohol consumption more common even among older people, the frequency of drinking (item 1) may be unrelated to the quantity items (2 and 3). This also applies to item 4 concerning how often the responders felt difficulties to stop drinking after a drinking session. Item 4 was shown to have a weak relation to the other items in the alcohol problems subscale.

The present findings cast doubt whether AUDIT is an appropriate instrument for screening problematic drinking among the elderly, it was clearly less reliable and valid in the elderly sample compared to the general population. To elucidate whether another method is more applicable, a comparison of different surveys between the age groups would be valuable. One suggestion is to clarify the items by presenting the AUDIT together with verbal explanations of the meaning of the items. Due to a higher incidence of cognitive impairment in older ages [4], this population may become intoxicated by a lower amount of alcohol than the general population suggesting that a lower cut-off score for older people should be tested as was recommended by Reinert & Allen [10] and O’Connell et al., [11]. A shorter version as AUDIT-5 may be valuable in this group. The optimal cut-off for risky drinking can be determined after such a study has been conducted. In the present study we did not set out to achieve this. In future studies it seems to be important to independently collect data about alcohol related harms and AUDIT data in elderly people to determine an appropriate cut-off.

### Limitations

A limitation of the study is the limited age range 79–80 years. In a forthcoming study we will expand the age span to include 70–80 year olds. Also the much wider age span (17–71) in the control group is a limitation due to the possibility of different factor structures within smaller age ranged (i.e. 17–25). A principal component analysis with varimax rotated components stratified on age showed that the 17–27 year olds and the 61–71 year olds displayed the 2-factor structure above. However the age group 28–60 years showed a fragmented 4-factor structure. This may explain the low fit shown in Table 2. However the sample was small when stratified on age groups making the components less stable. Another potential limitation is the percentage of non-responders, some of whom may be to ill or frail to respond.

## Conclusion

Compared to general population, the psychometric properties of AUDIT are not as good in a sample of elderly Swedish adults. Both the internal consistency reliability and the construct validity were weaker among older participants. This was mainly due to the weak reliability of item 1 and item 4 in older respondents. This finding should be considered when measuring alcohol habits in older populations.

## Competing interests

The authors declare that they have no competing interests.

## Authors’ contribution

HK is responsible for data collection, analyses and drafted the manuscript. PW read the draft, commented and made substantial contributions to the text. MR read and commented the early version of the manuscript and made substantial contributions to the text. MH read and commented the text and made substantial contributions to the manuscript. All authors read and approved the final manuscript.

## Pre-publication history

The pre-publication history for this paper can be accessed here:

http://www.biomedcentral.com/1471-2458/14/672/prepub
